# Attachment, Mentalization, and Criterion B of the Alternative *DSM-5* Model for Personality Disorders (AMPD)

**DOI:** 10.1186/s40479-021-00163-9

**Published:** 2021-08-02

**Authors:** Ericka Ball Cooper, Jaime L. Anderson, Carla Sharp, Hillary A. Langley, Amanda Venta

**Affiliations:** 1 Psychology Department, Sam Houston State University, Huntsville, TX USA; 2 NextSTEPS Worldwide, PLLC, McKinney, TX USA; 3 Psychology Department, University of Houston, Houston, TX USA

**Keywords:** Attachment, Mentalization theory, Personality disorders

## Abstract

**Background:**

The mentalization theory posits that interpersonal difficulties and maladaptive personality traits develop from an insecure attachment pattern with one’s caregiver and corresponding deficits in mentalizing—the ability to understand others’ and one’s own mental states. Mentalizing deficits have been theorized as the basis for all psychopathology, with the paradigmatic case being Borderline Personality Disorder. Nevertheless, developments in the personality field indicate personality pathology is best represented dimensionally, and such a proposal was outlined by the Alternative *DSM-5* Model for Personality Disorders (AMPD). Despite evidence linking the mentalization theory to personality disorders, however, it has yet to be applied to Criterion B of the AMPD. The aim of the present study was to evaluate the moderating role of mentalizing in the relation between attachment and Criterion B maladaptive trait function in a sample of undergraduates. We hypothesized a model in which: (1) attachment insecurity would be positively associated with the Negative Affectivity, Antagonism, and Disinhibition personality domains; (2) mentalizing ability would be negatively associated with these domains; and, (3) there would be an interaction effect between attachment and mentalizing when predicting these same domains.

**Methods:**

Personality domains were measured dimensionally via the Personality Inventory for *DSM-5* (PID-5-SF), while the dependence and avoidance domains of attachment were assessed via the Relationship Questionnaire (RQ). Mentalizing ability was tapped by the Movie for the Assessment of Social Cognition (MASC). The AMPD personality domains and trait facets were examined as dependent variables; attachment dependence, attachment avoidance, and overall mentalizing ability were entered as independent variables; and interaction terms between mentalizing and each attachment dimension were used to test moderation via MANCOVAs.

**Results:**

Consistent with expectations, results indicated overall mentalizing moderated the relation between attachment avoidance and Negative Affectivity. Posthoc analyses revealed similar effects on the relations between attachment avoidance and the Emotional Lability, Hostility, and Perseveration trait facets; however, there were no significant moderation findings related to attachment dependence.

**Conclusions:**

These results support the mentalization theory’s application to Criterion B of the AMPD, particularly in relation to the links between Negative Affectivity and borderline-related traits, and encourage future research of dimensional maladaptive personality. They further bolster support for understanding maladaptive personality as a dimensional construct.

The mentalization theory states that attachment (i.e., the internal working model one forms of themselves and others based on early caregiving experiences [1]) and mentalizing (i.e., understanding others’ and one’s own behavior as driven by underlying mental states) are key aspects in the development of interpersonal difficulties, maladaptive personality structures, and a range of negative psychopathological outcomes [2–4]. Indeed, the theory posits that disruptions in the attachment system, such as child maltreatment, thwart the development of accurate mentalizing abilities due to a lack of appropriate (marked) affective mirroring [3, 5]. It is theorized that these disruptions ultimately prevent the formation of a coherent structure of the self, resulting in disturbed identity formation, mentalizing, and interpersonal functioning [3]. More specifically, mentalization is proposed as a function of a child’s relationship with their caregiver, in which the messages that they are or are not receiving from their caregiver impact their ability to learn mentalizing skills, while their mentalizing skills also feed back into the child’s relationships with their caregiver and other attachment figures later on in life. For instance, an infant whose parent rarely interacts with them or maintains a lack of emotional connectedness with the child is also not providing feedback and information to the infant about the emotions they and the parent may be experiencing. This lack of emotional sharing results in the child not understanding how to interpret others’ or their own emotions, including their parent’s, leading to impaired self and interpersonal functioning [3, 5].

The literature on the mentalization theory largely supports mentalization’s role in a range of psychopathological disorders (e.g., eating disorders, substance use) [6, 7], though the model is most often associated with the development of pathological personality structures [3], including personality disorders [4, 8–10]. Many of the difficulties and symptoms associated with these disorders, particularly Borderline Personality Disorder (BPD), Antisocial Personality Disorder (ASPD), and Narcissistic Personality Disorder (NPD), are also reflected in the theorized outcomes of the mentalization theory, such as interpersonal difficulties, disruptions in attachment, and an unstable sense of self [3, 10–12]. Interestingly, Bateman and Fonagy [10, 13] argued that individuals with these personality disorders can present differently from one another when examining their behaviors from a mentalization standpoint. For instance, individuals with NPD tend to have a greater self-focus and decreased sense of others, while those with ASPD exhibit a reduced understanding of the self but better grasp on interpreting others [10, 13]. Additionally, individuals with BPD often fluctuate in their mentalizing capabilities, such that they can accurately mentalize in certain situations but “lose” this ability in others [13]. Despite these differences in mentalizing abilities across disorders, however, there is evidence to suggest there are deficits across the board, and that these deficits occur in accordance with activation of the attachment system (i.e., during interpersonal interactions), resulting in increased vulnerabilities to emotional state changes and impulsive behaviors among those with BPD, a concentration on one’s own mental states among individuals with NPD, and a focus on manipulating others among individuals with ASPD [13, 14].

Additional support for the link between maladaptive personality and the mentalization theory has been provided by clinical applications of this model. For instance, Bateman and Fonagy’s [10] Mentalization-Based Treatment (MBT) is a well-validated therapy modality, in which treatment focuses on the development of mentalizing skills within an interpersonal, attachment-driven context. MBT has demonstrated utility in reducing common borderline symptoms [10, 13, 15], and studies have supported its use among individuals with ASPD [10, 16] and NPD [17] as well. This research suggests that personality pathology shares a common etiological basis that is rooted within the attachment-mentalization paradigm, and that, by improving mentalizing abilities, one’s interpersonal and self-functioning can also improve [10, 13, 15–17]. Moreover, it supports a dimensional representation of personality—the idea that personality is structured according to a variety of domains and traits, rather than discrete disorders [18], and, indeed, MBT demonstrates effectiveness across several personality pathologies, suggesting that a lack of appropriate mentalizing abilities may be the common thread by which different personality presentations exhibit interpersonal difficulties. In fact, a plethora of research lends credence to a dimensional model of personality [19], necessitating analysis of the mentalization theory and its constructs (i.e., attachment and mentalizing) in the context of dimensional maladaptive traits. As such, and given the aforementioned commonalities across personality pathologies, the broad aim of this study was to examine the mentalization theory’s relation to underlying personality structures.

The most well-known dimensional model of maladaptive personality was proposed in the most recent edition of the *Diagnostic and Statistical Manual of Mental Disorders – Fifth Edition* (*DSM-5*) [11], titled the Alternative *DSM-5* Model of Personality Disorders (commonly referred to as the AMPD), in which personality dysfunction is defined through a moderate or greater level of impairment in personality functioning (Criterion A) and the presence of pathological personality traits (Criterion B) [11]. The AMPD proposes these “pathological traits” consist of 25 facets nested within one of five domains: Negative Affectivity, Antagonism, Detachment, Disinhibition, and Psychoticism (see Table 1) [20]. For instance, an AMPD diagnosis of BPD would require the demonstration of four or more maladaptive traits anomalous to BPD, in addition to identified functional impairment [11]. Research has demonstrated strong overlap in these trait facets and domains when comparing the AMPD and dimensional models of non-pathological personality, suggesting the domains correspondingly represent the maladaptive variants of normative personality structure [21–24].

**Table 1:** *DSM-5* Dimensional 25-Trait facet model [11, 20, 21]

Alternative Model Domain (Associated FFM Domain)	Brief Description	Pathological Trait Facets
Negative Affectivity (Neuroticism)	Wide range of negative emotions and associated behavioral manifestations experienced frequently, intensely, and at high levels	1. Anxiousness
		2. Emotional Lability
		3. Hostility
		4. Perseveration
		5. (Lack of) restricted affectivity
		6. Separation insecurity
		7. Submissiveness
Detachment (Extraversion)	Limited capacity for pleasure, avoidance of socioemotional experience, and withdrawal from others	8. Anhedonia
		9. Depressivity
		10. Intimacy avoidance
		11. Suspiciousness
		12. Withdrawal
Antagonism (Agreeableness)	Behaviors that put one at odds with others, such as high self-importance, and callous antipathy	13. Attention seeking
		14. Callousness
		15. Deceitfulness
		16. Grandiosity
		17. Manipulativeness
Disinhibition (Conscientiousness)	Impulsive behaviors driven by need for immediate gratification and without regard for consequences	18. Distractibility
		19. Impulsivity
		20. Irresponsibility
		21. (Lack of) rigid perfectionism
		22. Risk taking
Psychoticism (Openness to Experience^a^)	Odd, eccentric, or unusual behaviors/cognitions	23. Eccentricity
		24. Perceptual dysregulation
		25. Unusual beliefs/experiences

*Note.* ^a^Mixed findings regarding this link

Nevertheless, the existing literature has thus far not explored the application of the mentalization theory to the dimensional model of maladaptive traits; this is an important endeavor for several reasons. First, results consistent with our hypotheses would provide additional evidence for the AMPD, and a dimensional understanding of pathological personality more broadly, within the context of a well-researched etiological theory. In particular, this evidence would support the literature’s recent shift toward a dimensional view of personality disorders, as well as the clinical psychology field’s push to incorporate this model into the *DSM-5* and *International Classification of Disease, 11th edition* (ICD-11) [25]. Second, understanding how attachment and mentalizing relate to maladaptive personality traits can help identify individuals whose caregiving environment may place them at higher risk of developing these traits. Third, linking these constructs to maladaptive traits may support early intervention utilization for a wider client audience (e.g., MBT [10]).

Links between attachment, mentalizing, and pathological personality do exist in the literature, though only a small portion of this research has used Criterion B of the AMPD or similar models. The link between attachment and personality disorders, for instance, has been clearly documented: attachment styles have been associated with every *DSM-5* personality disorder, including ASPD and NPD [26, 27]. The link between insecure attachment patterns and BPD has been the primary focus, however [28–30]. For instance, secure attachments are much less common in adults with BPD (0–30%) than non-clinical samples [31]. Likewise, Bartholomew and Horowitz’s [32] model of attachment has been linked with the Five-Factor Model of personality (FFM) [19], such that the attachment dependence (i.e., the degree to which one feels worthy of love by others) and avoidance (i.e., one’s expectations of intimacy or closeness with others) dimensions have demonstrated positive associations with Neuroticism and negative associations with Extraversion, Agreeableness, and Openness to Experience [33, 34]. The dependence dimension is also negatively associated with Conscientiousness [33, 35].

Thus far, adult attachment has been linked with Criterion B of the AMPD in only one study [36], in which Fossati and colleagues [36] utilized the Attachment Styles Questionnaire (ASQ), a dimensional measure of attachment with five subscales that overlap with Bartholomew and Horowitz’s [32] dependence and avoidance model of attachment [37]. The ASQ scales predicted the five maladaptive personality domains, as well as 24 of the 25 maladaptive traits [36], demonstrating the first connection between Criterion B of the AMPD with the dependence and avoidance attachment domains. Still, the literature body currently lacks any further exploration of these relations, and, as such, replication is needed.

Mentalizing (also referred to as theory of mind, reflective functioning, and social cognition) is the ability to understand one’s own behavior, and the behavior of others, as guided by underlying mental states [13], and it has also been linked with personality pathology in extant literature. For instance, deficits in mentalizing have been associated with ASPD [16, 38, 39] and NPD^9, 17.^ Further, mentalizing has been identified as a translational construct in the conceptualization and treatment of BPD [40], and the disorder has been empirically and theoretically connected to a range of mentalizing difficulties (e.g., facial emotion recognition, hypermentalizing) across a number of studies and populations [14, 41–47]. For example, one study that explored the differences in reflective functioning abilities between adults with BPD and healthy controls indicated that the former group had more difficulty in both affective and cognitive theory of mind tasks [48]. Individuals with BPD also reported more difficulty with empathic reasoning, and their mentalizing errors were observed to coalesce around certain maladaptive attributions, such as black-and-white thinking [48].

Notably, several dimensional personality domains have also been linked with mentalizing; however, the research on these links is quite limited and usually constrained to the FFM domains, rather than the pathological variants, or between mentalizing and Criterion A of the AMPD. For instance, Nettle and Liddle [49] found that theory of mind was positively correlated with Agreeableness and negatively correlated with Neuroticism. A study conducted by Dimitrijević et al. [50] also yielded positive correlational results between mentalizing and Extraversion, Openness to Experience, and Conscientiousness, and a negative correlation with Neuroticism. Despite this evidence linking the FFM to mentalizing abilities, only two studies to the authors’ knowledge have explored relations between mentalizing and AMPD Criterion B constructs. In one study, Fossati and colleagues [39] found several associations between underlying trait facets and mentalizing abilities. More specifically, the Emotional Lability and Risk-taking trait facets were linked with hypomentalizing (that is, failing to attribute mental/emotional processes to others), while the Hostility, Suspiciousness, Withdrawal, Callousness, Deceitfulness, Lack of Rigid Perfectionism, and Unusual Beliefs and Experiences trait facets were correlated with hypermentalizing (i.e., overattributing mental or emotional processes to others) [39]. Additionally, da Costa and colleagues [51], found significant, negative correlations between overall mentalizing abilities and all five AMPD Criterion B domains.

Links between Criterion A of the AMPD (i.e., impairment in personality functioning) and mentalizing deficits have been explored [45, 52], further supporting the theoretical underpinnings of mentalization and maladaptive personality functioning being related. For example, Bender, Morey, and Skodol [52] explored personality dysfunction by examining a number of concepts to establish the Levels of Personality Functioning. Their findings demonstrated that mentalizing-related deficits, such as social cognition and reflective functioning, were related to impairment in personality functioning [52]. Nonetheless, neither of these studies, nor the aforementioned studies associating the FFM and mentalizing deficits, have explored the role of attachment in these relations. Additionally, no research has explored the potential application of the full mentalization theory (i.e., that attachment and mentalizing interact) to Criterion B of the AMPD at the time of this writing.

## The present study

Given prior research demonstrating the links between attachment, mentalizing, and personality [13–15, 42], the current study sought to explore the applicability of the mentalization theory to the AMPD. More specifically, we examined relations between attachment, mentalizing, and their interaction (i.e., such that mentalizing acts as a moderator) when predicting each pathological personality domain (i.e., Negative Affectivity, Antagonism, Detachment, Disinhibition, and Psychoticism [11]), as well as relevant demographic variables (i.e., gender, race/ethnicity, and age) as covariates. Indeed, our study sought to extend the findings of Fossati et al. [36, 39], by demonstrating the unique links between the AMPD’s Criterion B constructs with attachment and mentalizing while also exploring the potential moderating role of mentalizing on the relation between attachment and the AMPD. Importantly, mentalizing was used as a moderator given the aforementioned findings regarding there being differential behaviors and personality styles associated with various levels of mentalizing. Results supporting these links would assist in demonstrating the utility of the AMPD, particularly Criterion B, and encouraging the field’s shift toward a dimensional maladaptive personality. Additionally, results consistent with our expectations would encourage early identification of and intervention utilization for at-risk individuals (e.g., MBT [10]).

**Fig. 1:**
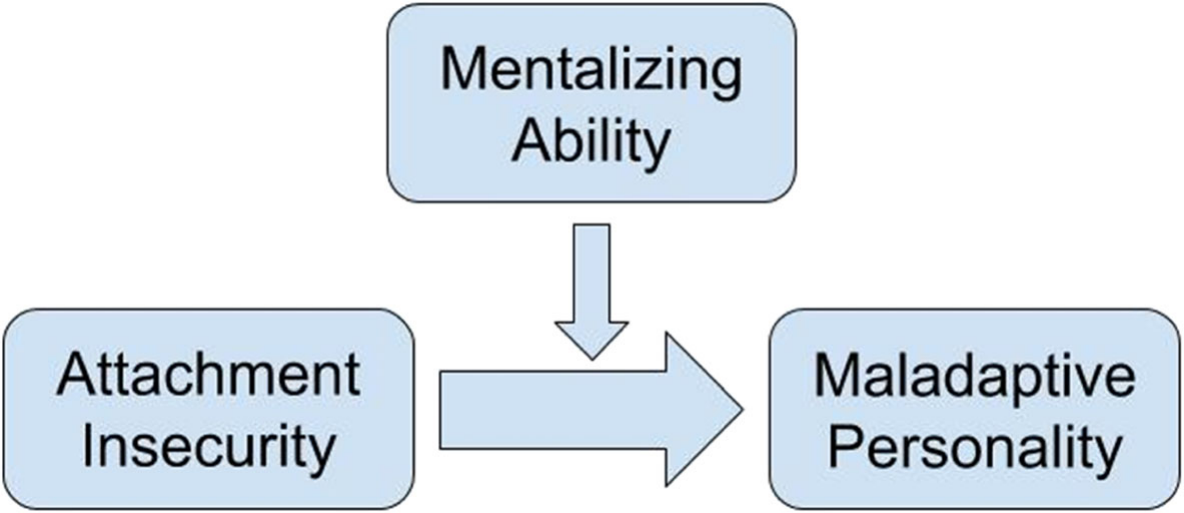
Proposed model regarding mentalizing as a moderator of attachment and maladaptive personality (e.g., AMPD domains)

To this end, we hypothesized a model in which:

1. the Negative Affectivity, Antagonism, and Disinhibition personality domains would be positively associated with attachment insecurity;
2. mentalizing ability would be negatively associated with each of these domains; and,
3. consistent with the mentalization theory of BPD, there would be an interaction effect between attachment and mentalizing when predicting domains commonly associated with borderline, antisocial, and narcissistic personality patterns (i.e., Negative Affectivity, Antagonism, and Disinhibition [11]; see Fig. 1 for our proposed model).

Notably, no specific hypotheses regarding the Detachment and Psychoticism domains were generated due to the paucity of research exploring their relation to the mentalization theory. Additionally, given that Criterion B of the AMPD lists several trait facets of Antagonism, Disinhibition, and Negative Affectivity within the new classifications of BPD, ASPD, and NPD [11], we hypothesized the links between one’s attachment and these personality domains would be moderated by mentalizing ability. Specifically, we proposed that individuals with low attachment security (i.e., high dependence or high avoidance) and less accurate mentalizing abilities would be significantly more likely to score higher on these maladaptive domains than those individuals with low attachment security and more accurate mentalizing abilities.

Finally, we conducted subsequent exploratory analyses to unpack findings for models that proved significant by exploring the mentalization theory’s application to the trait facets underlying each significant personality domain. For instance, we hypothesized that, should there be a significant interaction effect on the Negative Affectivity domain, analyses would be conducted on the seven trait facets underlying this domain (e.g., Separation Insecurity, Anxiousness [11]). Importantly, these analyses were exploratory in nature as the specific trait facets tested were yet to be determined at the time of hypothesis-generating.

## Method

### Participants

This study collected data from undergraduate psychology students at a university in the Southwestern United States. Indeed, a college-age sample is often quite useful in maladaptive personality research, as studies indicate that college students experience psychological distress at a higher rate than same-age peers not attending college [53]. This disparity in prevalence rates can also be found with personality disorders, as one study found that over 17% of college student participants displayed clinically significant symptoms of BPD [54], despite the prevalence in the general population being estimated at between one and six percent [11]. Furthermore, Trull [55] has argued that the level of personality dysfunction among college-age adults is clinically impairing. Attachment security is generally stable across the lifespan [56], and the transition between adolescence and young adulthood (such as when young adults first attend college) can be a particularly salient time to measure attachment-related variables given the changing dynamics of the parent-child relationship and need for independence [57].

Inclusion criteria for this study were an age of at least 18 years and English fluency, and data were collected as part of a larger study. Notably, a priori power analyses utilizing the G*Power Statistical Analysis tool for the larger study suggested a minimum sample size of 215 (given the following parameters: effect size > .15, α = .05, number of predictors = 3, and number of analyses = 5) [58, 59], and data were ultimately collected for a period of 1 month via an online portal system yielding a total sample size of 401 participants, 372 of whom completed all relevant measures. Data from seven participants were excluded due to participants taking fewer than 30 min to complete the survey, a timeframe identified by the data collectors as substantially shorter than the minimum amount of time to thoroughly watch all presented videos (i.e., 15 min or longer) and consider all presented questions (approximately 315). Descriptive statistics supported this cut-off time, as the dataset’s median completion time was 76 min. Additionally, 26 participants’ responses were excluded from the dataset in accordance with validity cut-offs described in the validity measure section. Given that gender group was used as a covariate in later analyses, and only one participant identified as non-binary, this participant’s data was excluded from later analyses. The final subsample consisted of 338 participants.

Participants ranged in age from 18 to 45, with a median age of 18. Most participants (95.9%; *n* = 324) fell between the ages of 18 and 23. The sample was largely female (*n* = 292; 86.4%) and reported being single and never married (*n* = 321; 95.0%). Most participants identified as White, Black or African American, or Hispanic or Latino (*n* = 323; 95.6%), and due to the small number of participants identifying as another race or ethnicity, three racial/ethnic groups were collapsed into an “other” group (*n* = 15, 4.4%) in order to add race/ethnicity as a covariate into later analyses. Further statistics regarding demographic variables can be observed in Table 2.

**Table 2:** Statistics for demographic variables utilized in later analyses

Study participants (*N* = 338)	
Median Age	18 years (Range: 18–45)
Gender	
Female	292 (86.4%)
Male	46 (13.6%)
Race/Ethnicity	
Asian	8 (2.4%)
American Indian/Alaskan Native	5 (1.5%)
Black/African American	54 (16.0%)
Hispanic/Latino	119 (35.1%)
Native Hawaiian/Pacific Islander	2 (0.6%)
White	150 (44.4%)

### Measures

#### Demographic information

Participants were queried regarding basic demographic information, namely their age, gender, race/ethnicity, and marital status. Participants were asked to enter a numerical value for their age; however, all other questions were forced choice (e.g., “yes” and “no” response options for Hispanic or Latino ethnicity).

#### Maladaptive personality

The Personality Inventory for *DSM-5* Short Form (PID-5-SF) is a 100-item self-report inventory that assesses the 25 pathological personality trait facets and five domains of personality [60]. It was developed via item response theory as a shortened version of the PID-5 [61]. Similar to the original version, participants rated items on a four-point Likert-type scale from 0 (very/often false) to 3 (very/often true), and subscale scores are obtained by summing individual items, with some items being reverse scored. Although the PID-5-SF is not used as widely as the original version, the literature body supports its use, and studies have demonstrated adequate reliability and validity across populations [60, 62, 63]. For the present study, all major scales demonstrated adequate internal consistency (Negative Affectivity: α = .91; Detachment: α = .90; Antagonism: α = .85; Disinhibition: α = .86; Psychoticism: α = .86).

#### Attachment

The Relationship Questionnaire (RQ) [32] was used to assess participants’ attachment security via a forced-choice instrument, such that it provides descriptions of four attachment styles (i.e., secure, dismissing, preoccupied, and fearful) and asks participants to select which description sounds most similar to their own relationships (one item). Additionally, the measure asked participants to rate how well each of the four styles described them on a Likert-type scale from 1 (disagree strongly) to 7 (agree strongly), and, as such, it can provide dimensional measures of avoidance and dependence [37]. The RQ was chosen for our study due to its brevity, its ability to categorically and dimensionally describe attachment, and its demonstrated reliability and validity among adult and community populations [32]. In keeping with the dimensional ideals we used for personality and mentalizing, we utilized the RQ’s avoidance and dependence dimensions. Internal consistency was not computed for this measure, which is not used in a continuous manner.

#### Mentalizing

The Movie for the Assessment of Social Cognition (MASC) was utilized to measure participants’ mentalizing abilities. The MASC is a video-based instrument consisting of a 15-min long film, which is stopped at various points to ask a total of 45 questions about the character’s mental states [14, 42, 64]. Each question provided four response choices that represent different levels of mentalizing ability (i.e., no mentalizing, hypomentalizing, accurate mentalizing, and hypermentalizing). Participants’ responses were summarily scored within each response category. An overall mentalizing score was obtained by subtracting the number of errors from the accurate mentalizing total, wherein a higher final score indicated more accurate mentalizing. The MASC has demonstrated high reliability and validity among clinical and community populations and is often considered to be the gold standard of social cognition measures due to its objective measure of mentalizing abilities [14, 42, 64]. The internal consistency for our sample was acceptable (α = .74), consistent with prior studies [36, 64].

#### Validity

To ensure the validity of participants’ responses on the personality and attachment measures, control items were added to the administration of the larger study. A total of eight items was used across the administration and consisted of nonsensical or illogical statements. Response styles corresponded with the measure in which the validity question was included. For instance, given that the PID-5-SF requires participants to answer on a scale from 0 (very false or often false) to 3 (very true or often true), a control item for that measure asked participants to rate the statement, “When I see the color orange, I taste mustard,” on a scale from 0 to 3. Validity items within the attachment measure also reflected that scale’s specific response style. As mentioned earlier, the data for those participants who provided two or more invalid responses were excluded from analysis (*n* = 26), due to most participants having zero or one invalid response.

### Procedure

This study was approved by the appropriate institutional review board prior to data collection. It was posted on a data collection website specifically for undergraduate students, such that students selected to engage in the study for academic credit and anonymously completed the battery of measures online. Informed consent was obtained, and demographic information was then acquired, followed by the PID-5-SF, RQ, and MASC measures. The data were de-identified prior to analysis.

## Results

### Preliminary analyses

Preliminary tests analyzing normality and heteroscedasticity (i.e., histograms, skewness and kurtosis tests, and scatterplots) suggested three of the PID-5-SF scales were significantly negatively skewed. As such, square root transformations were conducted on the Detachment, Psychoticism, and Antagonism scales. The Negative Affectivity and Disinhibition scales appeared to be normally distributed.

Demographic data were analyzed via *t*-tests, one-way Analyses of Variance (ANOVAs), and correlational analyses to determine if there were any relations between gender, race/ethnicity, and age with key study variables (i.e., personality, attachment, and mentalizing). These analyses indicated the relations between gender and Negative Affectivity [*t*(335) = 3.99, *p* < .001], ethnicity group and Disinhibition, [*F*(3, 334) = 3.32, *p* = .02], and age and overall mentalizing were significant [*r*(338) = .11, *p* = .04]. More specifically, female participants endorsed significantly more items relating to Negative Affectivity, Hispanic participants endorsed significantly more Disinhibition items than White participants, and older participants demonstrated more accurate mentalizing abilities. Given these differences, gender, race/ethnicity group, and age were included as covariates. Additional statistics can be found in Table 3.

**Table 3:** Preliminary Analyses amongst Demographic and Key Study Variables

Study Participants (*N* = 338)				
Scale	Gender (*t*)	Ethnicity (*F*)	Age (*r*)	Mean (SD)
PID-5-SF Negative Affectivity	3.99***	0.29	−.08	1.33 (0.53)
PID-5-SF Detachment	1.17	1.71	−.07	0.76 (0.33)
PID-5-SF Antagonism	−1.47	0.22	−.01	0.64 (0.28)
PID-5-SF Disinhibition	0.85	3.32*	−.10	0.84 (0.43)
PID-5-SF Psychoticism	0.84	1.03	−.10	0.73 (0.40)
RQ Dependence	1.84	2.08	−.05	0.67 (5.08)
RQ Avoidance	1.37	2.29	.02	0.65 (4.40)
MASC Overall	0.17	2.11	.11*	17.40 (10.63)

*p < .05, **p < .01, ***p < .001

Note. PID-5 -SF = Personality Inventory for DSM-5—Short Form; *RQ =* Relationship Questionnaire, *MASC =* Movie for the Assessment of Social Cognition

### Multivariate analyses

Multivariate Analyses of Covariance (MANCOVAs) were utilized to test our hypotheses, due to this method’s parsimonious ability to analyze main and interaction effects on multiple dependent variables while simultaneously controlling for covariates and family-wise error. Indeed, this method allowed us to explore the main effects proposed in our first and second hypotheses (i.e., attachment security and mentalizing would be negatively associated with the Negative Affectivity, Disinhibition, and Antagonism domains), as well as the moderating effect proposed by our third hypothesis. Two MANCOVAs were conducted, one for attachment dependence and another for attachment avoidance. Each analysis included all personality domains (i.e., Negative Affectivity, Detachment, Antagonism, Disinhibition, and Psychoticism) as dependent variables; the relevant attachment dimension (dependence or avoidance), overall mentalizing score, and their interaction as independent variables; and age, ethnicity, and gender as covariates. Multivariate results of the attachment dependence MANCOVA indicated a significant main effect of attachment dependence on personality (see Table 4). At the univariate level, dependence was associated with all five domains, most notable being Negative Affectivity. There were no significant multivariate main effects of mentalizing, nor was the interaction term significantly related to any personality domain.

**Table 4:** MANCOVA Results of attachment dependence and overall mentalizing on the PID-5-SF personality domains

	Wilk’s Λ	*F*	df	*p*	*η*^*2*^
Multivariate Results					
Intercept	.74	22.63***	5, 325	< .001	.26
Gender	.94	4.09**	5, 325	.001	.06
Ethnicity	.92	1.88*	15, 898	.02	.08
Age	.99	1.01	5, 325	.41	.01
RQ Dependence	.92	5.85***	5, 325	< .001	.08
MASC	.98	1.53	5, 325	.18	.02
RQ x MASC	.99	0.75	5, 325	.59	.01
Univariate Results					
Negative Affectivity	–	23.88***	1, 329	< .001	.07
Detachment	–	15.12***	1, 329	< .001	.04
Antagonism	–	4.46*	1, 329	.04	.01
Disinhibition	–	7.36*	1, 329	.01	.02
Psychoticism	–	7.61***	1, 329	< .001	.02

*p < .05, **p < .01, ***p < .001

Note. PID-5-SF = Personality Inventory for DSM-5—Short Form; *RQ =* Relationship Questionnaire, *MASC* = Movie for the Assessment of Social Cognition

**Fig. 2:**
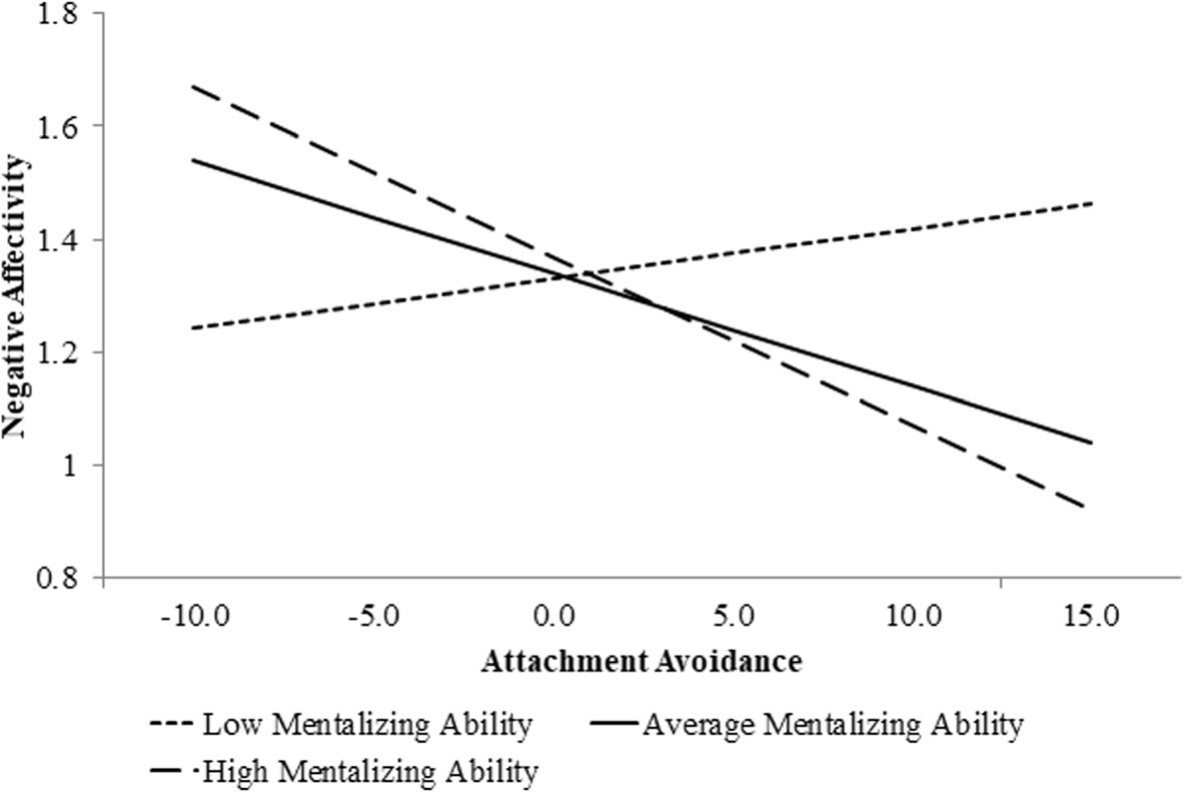
Simple slopes of attachment avoidance predicting negative affectivity for one *SD* below/above and at the mean of mentalizing

In the second model, neither attachment avoidance nor overall mentalizing had a significant main effect on personality. However, a significant moderating effect by overall mentalizing was observed at the multivariate level (see Table 5). Univariate analyses indicated a significant moderation of the Negative Affectivity domain, such that participants who scored low on overall mentalizing and high on attachment avoidance also rated themselves as having more negative affect than individuals with average or high mentalizing abilities with high avoidance (see Fig. 2). No moderating effects were observed for the Detachment, Antagonism, Disinhibition, or Psychoticism domains in relation to attachment avoidance.

**Table 5:** MANCOVA Results of attachment avoidance and overall mentalizing on the PID-5-SF personality domains

	Wilk’s Λ	*F*	df	*p*	*η*^*2*^
Multivariate Results					
Intercept	.77	18.97***	5, 325	< .001	.77
Gender	.91	6.44***	5, 325	< .001	.09
Ethnicity	.92	1.79*	15, 898	.03	.08
Age	.98	1.32	5, 325	.26	.02
RQ Avoidance	.98	1.58	5, 325	.17	.02
MASC	.97	1.82	5, 325	.11	.03
RQ x MASC	.95	3.21*	5, 325	.01	.05
Univariate Results					
Negative Affectivity	–	3.99*	1, 329	.04	.01
Detachment	–	2.31	1, 329	.13	.01
Antagonism	–	0.45	1, 329	.50	.00
Disinhibition	–	1.76	1, 329	.19	.01
Psychoticism	–	0.41	1, 329	.52	.00

*p < .05, **p < .01, ***p < .001

Note. PID-5 -SF = Personality Inventory for DSM-5—Short Form; *RQ =* Relationship Questionnaire, *MASC =* Movie for the Assessment of Social Cognition

### Posthoc analyses

Consistent with our proposed aims to explore the maladaptive personality model in greater depth, and our third hypothesis being supported in relation to attachment avoidance and the Negative Affectivity domain, posthoc analyses were conducted for the seven trait facets underlying this domain. Thus, we explored each trait facet (i.e., Anxiousness, Emotional Lability, Hostility, Perseveration, Lack of Restricted Affectivity, Separation Insecurity, and Submissiveness) as a dependent variable in relation to attachment avoidance and overall mentalizing. Notably, attachment dependence was not explored, given the lack of statistically significant findings between this attachment dimension and Negative Affectivity. Attachment avoidance, overall mentalizing, and their interaction were therefore maintained as independent variables, while age, ethnicity, and gender remained as covariates. The trait facets were examined for potential violations of normality and homoscedasticity. The Emotional Stability and Hostility subscales appeared to be significantly negatively skewed and were subsequently transformed using the square root function. The Lack of Restricted Affectivity subscale was positively skewed and transformed by squaring the variable.

Results demonstrated a significant multivariate main effect of attachment avoidance. When examining the results of univariate analyses, attachment avoidance had significant main effects on the Hostility and Lack of Restricted Affectivity subscales; no such effect was observed with overall mentalizing. Interaction effects proved significant at the multivariate level (Table 6), as well as with three of the trait facets: Emotional Lability, Hostility, and Perseveration. That is, those participants who reported lower mentalizing ability and higher attachment insecurity also endorsed experiencing more negative affect in these specific areas. No interaction effects were observed for the Anxiousness, Lack of Restricted Affectivity, Separation Insecurity, and Submissiveness subscales.

**Table 6:** MANCOVA results of attachment avoidance and overall mentalizing on the PID-5-SF Negative Affectivity trait facets

	Wilk’s Λ	*F*	df	*p*	*η*^*2*^
Multivariate Results					
Intercept	.77	13.62***	7, 323	< .001	.23
Gender	.84	8.51**	7, 323	< .001	.16
Ethnicity	.94	1.00	21, 928	.47	.06
Age	.93	3.52**	7, 323	.001	.07
RQ Avoidance	.92	4.07***	7, 323	< .001	.08
MASC	.98	1.04	7, 323	.40	.02
RQ x MASC	.95	2.70*	7, 323	.01	.05
Univariate Results					
Anxiousness	–	0.89	1, 329	.35	.00
Emotional Lability	–	3.66*	1, 329	.05	.01
Hostility	–	4.83*	1, 329	.03	.01
Perseveration	–	4.65*	1, 329	.03	.01
(Lack of) Restricted Affectivity	–	1.38	1, 329	.24	.00
Separation Insecurity	–	.39	1, 329	.53	.00
Submissiveness	–	.21	1, 329	.65	.00

*p < .05, **p < .01, ***p < .001

Note. PID-5 -SF = Personality Inventory for DSM-5—Short Form; *RQ =* Relationship Questionnaire, *MASC =* Movie for the Assessment of Social Cognition

## Discussion

The present study was designed to assess the unique links between attachment, mentalizing, and the AMPD personality domains, as well as to explore mentalizing ability as a moderator of the relation between attachment and the maladaptive personality domains. In particular, we proposed that this moderation would be significant in relation to Negative Affectivity, Antagonism, and Disinhibition because of their inclusion in the diagnoses of BPD, ASPD, and NPD [11]. Results provided mixed support for our hypotheses.

As predicted, we found a significant moderating effect for overall mentalizing on the association between attachment avoidance and the Negative Affectivity domain, such that participants high on attachment avoidance and with less accurate mentalizing abilities endorsed higher Negative Affectivity than those individuals with similar attachment avoidance scores but more accurate mentalizing abilities. Post-hoc analyses supported the interaction of mentalizing ability and attachment avoidance for the Emotional Lability, Hostility, and Perseveration trait facets of Negative Affectivity, but not for the remaining facets (i.e., Anxiousness, Lack of Restricted Affectivity, Separation Insecurity, and Submissiveness).

These findings are broadly consistent with the wider literature supporting the mentalization theory, particularly when examining the vast body of research connecting the model to BPD [3, 10]—a disorder marked by pervasive difficulties with mood lability, hostile behaviors, and poor interpersonal relationships [11]. The results also lend support to the application of the mentalization theory to the alternative *DSM-5* model trait profile for ASPD, given that Hostility is one of the pathological personality traits proposed to underlie the disorder [11], and previous research has found utility in treating ASPD patients with Mentalization-Based Treatment (MBT) [10, 13, 15, 16]. The current study thus extends this literature base to the AMPD, suggesting the mentalization theory applies to individuals with high Negative Affectivity, regardless of diagnostic classification. Additionally, our study supports prior research indicating mentalizing ability acts as a transdiagnostic mechanism and that, by providing MBT for a wide range of diagnoses (e.g., eating disorders and substance use disorders [6, 7, 13]), the mechanism of change in symptom reduction is through mentalizing ability.

Notably, although we anticipated the remaining trait facets of Negative Affectivity would also have significant moderations, it may be that these findings were not demonstrated given that the AMPD, and each individual domain, are constructed from a mix of behaviors, affects, and perceptions. That is, while mentalization [3, 5] and attachment [1] theories are driven by theory and compose a coherent concept, the AMPD’s individual domains and trait facets are empirically-based and describe different components of the higher-level construct [11]. Subsequently, the other facets that compose the Negative Affectivity domain may have limited overlap with those moderated by mentalizing.

Interestingly, evidence of significant moderation was not found for the Antagonism or Disinhibition domains, in contrast to prior research supporting the application of the mentalization theory to NPD and ASPD, disorders primarily composed of traits related to these domains [11]. Notably, however, BPD was the disorder on which the mentalization theory was originally theorized [2], and the model has only been applied to other personality pathologies more recently [9, 15, 17]. As such, the research connecting the mentalization theory to NPD and ASPD is relatively limited, and, particularly with NPD, is based more on theoretical underpinnings. The only study to our knowledge that has empirically explored the mentalization theory’s application to NPD did so in a sample of individuals with comorbid BPD [17].

Subsequently, it may be that the mentalization theory is only related to the Negative Affectivity aspect of maladaptive personality, resulting in the model being most closely linked with a disorder that is often associated with Negative Affectivity—BPD (*r* = .81 between BPD and Negative Affectivity in Fowler et al. [65]). Interestingly, the heterogeneity in the categorical diagnosis of BPD is quite extensive, given that there are 256 different ways to be diagnosed with BPD according to the *DSM-5*’s diagnostic criteria; however, the AMPD criteria for BPD primarily center on the trait facets that compose the Negative Affectivity domain [11, 66]. Furthermore, it may be that what was previously conceptualized as the mentalization theory of BPD is more accurately described as the mentalization theory of *Negative Affectivity*. This new conceptualization of the mentalization theory’s link to psychopathology warrants future research into the application of the model to any disorder that has a strong Negative Affectivity component, particularly those with symptoms related to Hostility, Emotional Lability, and Perseveration.

Another unexpected finding from our study is the presence of a significant moderation of mentalizing ability when examining attachment avoidance, but not dependence. Given that our study is the first to apply the mentalization theory to dimensional personality, there are no other studies that can fully support or oppose our findings; however, studies using dimensional attachment constructs when examining the mentalization theory and BPD have thus far provided mixed results. For instance, although some studies have found links only between the avoidance dimension and mentalizing [36, 67], others have demonstrated similar findings with only attachment dependence [68, 69]. One potential explanation for this difference in findings is the specific mentalizing ability tapped in these studies as compared to our project. More specifically, while the tasks used in the dependence-supporting studies used measures that examined mentalizing abilities for the self and others (i.e., the Metacognition Assessment Scale—Abbreviated [68] and the Mental States Task [69]), our mentalizing measure, the MASC, asks participants to hypothesize about fictional characters’ emotional and mental states [64]. Indeed, individuals high on attachment avoidance are inherently characterized by avoiding close contact with others, and, as such, it may be that the MASC is more sensitive to detecting mentalizing errors among those high in avoidance, but less useful in perceiving errors among high-dependence individuals (i.e., individuals who often look to others for validation [32]). Mentalizing ability should therefore be examined as a moderator again within the context of attachment and dimensional maladaptive personality, in which a self-focused mentalizing task is used.

Although our primary aim related to the mentalization theory (and moderation analyses), we also sought to examine main effects of attachment insecurity on the Negative Affectivity, Antagonism, and Disinhibition domains, expecting that higher rates of attachment dependence or avoidance would be positively associated with these domains. Our results supported this hypothesis when examining attachment dependence and each of the personality domains. Significant, positive associations were also found when examining the trait facets underlying the Negative Affectivity domain. These main effects of attachment dependence are consistent with previous research [33, 34], including the AMPD domains and 24 of 25 trait facets [36]. Our study also extends these findings to a diverse sample of American undergraduate students, as previous studies used samples of Swedish students [34] and Italian adults [36].

Contrary to our expectations, our findings did not provide evidence for main effects of attachment avoidance on Negative Affectivity, Antagonism, or Disinhibition domains. Previously, only one study has demonstrated a link between attachment avoidance and the AMPD Criterion B domains, and did so with a sample of Italian adults [36]. Although attachment is considered to be stable across cultures [70, 71] and the lifespan [56], it may be that these factors moderate the relation between attachment avoidance and personality, such that the level of avoidance is different during young adulthood than later in adulthood. Because many of our participants were enrolled in a fall-semester introductory psychology class and the median age was 18, it is likely this was their initial semester of college and first time away from home. Importantly, past research has indicated this time of life is marked by increased levels of *separation-individuation*, a normative developmental process during which young adults begin to separate themselves from parents to form a more coherent and autonomous self-identity [72]. As such, our sample’s attachment avoidance distribution may have been higher on average and had less variability than the Fossati et al. [36] sample because of their differing developmental stages. Nevertheless, additional research is needed to confirm this.

Lastly, we did not find support for our hypothesized main effects of mentalizing ability on Negative Affectivity, Antagonism, and Disinhibition, in contrast to prior research linking mentalizing errors to personality pathology, particularly BPD [40, 47]. Still, the previous literature demonstrating relations between mentalizing and *dimensional* personality is much more limited [49, 50], especially when examining maladaptive domains and trait facets [39, 51]. Previous studies utilized correlational methods [39, 51] when examining mentalizing pathological personality, rather than multivariate analyses like our study. Although our study conducted a priori power analyses, we utilized a more complex model that may have failed to detect small effects found in prior research, particularly given that many of our analyses were exploratory in nature. Subsequently, replication is needed to confirm the findings reported herein and avoid conclusions that have inadvertently capitalized upon Type I error or sample anomalies.

Nevertheless, it may be that mentalizing is indeed related to personality pathology but in relation to Criterion A of the AMPD. Indeed, such a link was recently demonstrated by Zettl and colleagues [73], in which all domains of the Level of Personality Functioning Scale (LPFS; a self-report tool that measures the level of impairment one is experiencing in their personality functioning) were significantly correlated with mentalizing abilities, suggesting a strong overlap between these constructs. Other studies have also demonstrated links between constructs of mentalizing and Criterion A of the AMPD [45, 52], as well as severity of borderline traits [68]. Future studies exploring level of impairment, as well as the four aspects of impairment (i.e., Identity, Self-Direction, Empathy, and Intimacy) should therefore be undertaken to determine if the mentalization theory in fact moderates one’s severity of impairment related to attachment security, rather than level or presence of traits.

This study need not be considered without limitation. Notably, these analyses were conducted from cross-sectional data and causal inferences cannot be made. Though a longitudinal study would still not be able to support causal relations (given our inability to manipulate variables like attachment security and mentalizing ability), longitudinal analyses using these same constructs could be helpful in determining if mentalizing moderates the relation between attachment and personality across the lifespan. Furthermore, response style biases and shared method variance cannot be eliminated as a possibility for self-report measures (e.g., the PID-5-SF, RQ). Future studies should attempt to collect data via non self-report approaches, such as the Adult Attachment Interview (i.e., the gold standard in assessing adult attachment style) or observational methods, in order to reduce potential sources of statistical noise. Lastly, although our sample of undergraduate students displayed adequate variability on the personality measures, several of the domains and trait facets, such as Psychoticism and Antagonism, were negatively skewed (i.e., most participants reported themselves to have low levels of these traits). Subsequently, our hypotheses should also be tested within a clinical sample, a setting wherein maladaptive personality traits are observed more often and could provide greater variability in personality-related variables. Notwithstanding these limitations, the present study expands the current evidence base regarding relations between the AMPD Criterion B constructs, attachment, and mentalizing ability to a diverse sample of undergraduate students.

## Conclusion

In sum, given that no other study has explored the application of the mentalization theory to dimensional, maladaptive personality, particularly Criterion B of the AMPD, our results are the first of their kind and indicate that mentalizing ability does, in fact, moderate the association between attachment and Negative Affectivity; however, it does so in relation to the attachment avoidance dimension only. More specifically, the present study established that individuals high on attachment avoidance and with less accurate mentalizing abilities rated themselves as experiencing more negative affectivity than those individuals with similar attachment avoidance scores but higher mentalizing abilities. These findings were also demonstrated with three of the seven trait facets underlying the Negative Affectivity domain: Emotional Lability, Hostility, and Perseveration. Nevertheless, inconsistent with our hypotheses, mentalizing was not found to moderate the Antagonism and Disinhibition domains, nor did it moderate Psychoticism and Detachment. Still, our findings support the mentalization theory’s application to Criterion B of the AMPD, and a dimensional understanding of pathological personality more broadly, as well as the use of MBT, particularly given the links between Negative Affectivity and BPD [11, 65]. Indeed, the current study’s results stand to inform intervention protocol, as they suggest that MBT would be particularly useful for individuals who frequently experience mood lability, hostility, or perseverating thoughts, in addition to decreased mentalizing abilities. The impact of our study, therefore, lies in identifying individuals who experience negative affect, regardless of their diagnosis, with the aim of reducing their symptoms via improved mentalizing abilities.

## Data Availability

The data that support the findings of this study are available on request from the corresponding author EBC. The data are not publicly available due to them containing information that could compromise research participant privacy/consent.

## Abbreviations

BPD: Borderline Personality Disorder
AMPD: Alternative Model of Personality Disorders
ASPD: Antisocial Personality Disorder
NPD: Narcissistic Personality Disorder
APA: American Psychiatric Association
MBT: Mentalization-Based Treatment
*DSM-5*: *Diagnostic and Statistical Manual of Mental Disorders – Fifth Edition*
*ICD-11*: *International Classification of Disease, 11th edition*
FFM: Five Factor Model
ASQ: Attachment Styles Questionnaire
PID-5: Personality Inventory for *DSM-5*
PID-5-SF: Personality Inventory for *DSM-5* Short Form
RQ: Relationships Questionnaire
MASC: Movie for the Assessment of Social Cognition
ANOVAs: Analyses of Variance
MANCOVAs: Multivariate Analyses of Covariance
LPFS: Level of Personality Functioning Scale
